# The cytokine profile of human NKT cells and PBMCs is dependent on donor sex and stimulus

**DOI:** 10.1007/s00430-016-0449-y

**Published:** 2016-02-20

**Authors:** Hannah Bernin, Helena Fehling, Claudia Marggraff, Egbert Tannich, Hannelore Lotter

**Affiliations:** Department of Molecular Parasitology, Bernhard Nocht Institute for Tropical Medicine, Bernhard-Nocht-Str. 74, 20359 Hamburg, Germany

**Keywords:** Sex difference, Immune response, NKT cells, Human, αGalCer, *Entamoeba histolytica*, Cytokines, Glycolipid

## Abstract

Sex-related variations in natural killer T (NKT) cells may influence immunoregulation and outcome of infectious and autoimmune diseases. We analyzed sex-specific differences in peripheral blood NKTs and peripheral blood mononuclear cells (PBMCs) from men and women and determined the frequencies of NKT cells and their subpopulations [CD4^+^; CD8^+^; double negative (DN)] and the levels of cytokine production following stimulation with the NKT cell ligands α-Galactosylceramide (αGalCer) and *Entamoeba histolytica* lipopeptidephosphoglycan (Lotter et al. in PLoS Pathog 5(5):e1000434, 2009). Total and DN NKT cells were more abundant in women than in men. In women, αGalCer induced higher production of intracellular IFNγ, IL-4, IL-17 and TNF by CD4^+^ and DN^+^NKT cells. Both ligands induced expression of multiple cytokines in PBMCs and influenced the ratio of NKT cell subpopulations during long-term culture. Although the sex-specific differences in frequencies of NKT cells and their subpopulations were marginal, the significant sex-specific differences in cytokine production might influence disease outcomes.

## Introduction

Immune responses differ between the sexes. In addition to behavioral, genetic, and hormonal factors, differences in the abundance and activation of various types of immune cells could explain some of the observed sexual dimorphisms in infectious diseases [2, 3]. Sex-specific differences in immune responses of women and men could underlie the higher susceptibility of men to infectious diseases caused by bacteria, viruses and parasites, e.g., tuberculosis [4], influenza A [5] and amebiasis [6–8]. By contrast, women exhibit more vigorous humoral and cellular immune responses and are more susceptible to cell-mediated autoimmune diseases [9, 10].

The relative proportions of certain immune cell populations differ between men and women. Men have higher monocyte counts [2] but a lower percentage of T lymphocytes within the total lymphocyte population [10]. A rare T cell subset, the invariant natural killer T (NKT) cells, also exhibits sex-related differences in frequency and is more abundant in women than in men [11–13]. NKT cells have immunomodulatory properties, bridge the innate and adaptive immune response and play crucial roles in a variety of infectious diseases, autoimmune disorders and cancers [14–16]. They express an invariant T cell receptor (TCR) that consists of the Vα24-Jα18/Vβ11 chains in humans and Vα14-Jα18/Vβ8.2 chains in mice. Based on their expression of CD4 and CD8, NKT cells can be divided into three subpopulations: CD4^+^, CD8^+^ and CD4^−^CD8^−^ (double negative; DN) [14]. Whereas conventional T cells recognize protein antigens in the context of MHC class I and II molecules, NKT cells recognize exclusively glycolipid and lipid antigens presented by the MHC I-like molecule CD1d [17]. Activated NKT cells can produce different pro- and anti-inflammatory cytokines, including interferon (IFN) γ, tumor necrosis factor α (TNF), interleukin (IL)-2, IL-4, IL-17, and tumor growth factor (TGF) β [18–20]. The strongest NKT cell activator identified to date is α-Galactosylceramide (αGalCer), a lipid molecule originally isolated from a marine sponge [21, 22]. Although NKT cells are attractive targets for immunotherapies, the very strong activation by αGalCer limits the clinical utility of this compound. Therefore, more moderate ligands provided by other microorganisms should be considered [23].

We recently isolated a glycolipid molecule from the membrane of a protozoan parasite, the *Entamoeba histolytica* lipopeptidephosphoglycan (*Eh*LPPG). *Eh*LPPG induces substantial IFNγ production in murine NKT cells in a CD1d-dependent manner, and treatment of mice with *Eh*LPPG considerably reduces abscesses in a murine model of hepatic amebiasis [1].

NKT cells are modulated by sex hormones such as 17β-estradiol and testosterone, leading to sex-specific differences in NKT-mediated immune responses. For example, treatment of mice with αGalCer increases the serum IFNγ levels in females, but not in males. This difference is absent in estrogen receptor-deficient mice or ovariectomized mice [24]. By contrast, testosterone substitution lowers the female resistance to hepatic amebiasis by decreasing IFNγ production in NKT cells [25].

In this study, we measured the frequencies of NKT cell and their subpopulations in men and women and analyzed the cytokines by stimulation with αGalCer and *Eh*LPPG. We also analyze the cytokine induction also in bystander cells and investigated the influence of αGalCer and *Eh*LPPG on NKT cell subpopulations upon enrichment.

## Materials and methods

### Human subjects and isolation of PBMCs

Peripheral blood mononuclear cells (PBMCs) from buffy coats or fresh blood samples from blood donors [23 men (31–53; 43.7 years ±5.8] and 22 women [33–51; 42.7 years ±6.1)] were used for cytokine analysis of NKT cells following stimulation with αGalCer and *Eh*LPPG. The average age of the blood donors ranged between 31 and 54 years (43.3 ± 5.8). Samples were subjected to flow cytometer, ELISA or used to generate APCs from CD14^+^ monocytes. Buffy coats for isolation of PBMCs were kindly provided by the Department of Transfusion Medicine of the University Clinic Hamburg-Eppendorf. The cohort size is indicated individually for each experiment. All experiments were approved by the ethical review committee of the medical council of Hamburg (PV3551).

Briefly, PBMCs were isolated by density-gradient centrifugation in Biocoll (Biochrom AG). The leukocyte ring was removed and washed twice with PBS. The resultant pellet was resuspended in 1 ml of X-VIVO™ 15 (LONZA) supplemented with 1 % Pen/Strep (AppliChem Panreac) or in RPMI 1640 (10 % FCS, 1 % l-Glutamine, 1 % Pen/Strep). PBMCs were then used in human NKT cell or stimulation assays or for generation of APCs.

### Intracellular cytokine production in human NKT cells

The NKT cell cytokine assay was a modification of a protocol described by Sandberg et al. [11]. In brief, 1 × 10^6^ human PBMCs/well were cultured in quadruplicates in 96-well round-bottom plates in X-VIVO™ 15 (LONZA) supplemented with 1 % Pen/Strep (AppliChem Panreac). Cells were stimulated with 1 µg/ml αGalCer or 10 µg/ml purified *Eh*LPPG. To achieve optimal NKT cell activation without additional APCs, 3 µg/ml purified αCD28 was added to each well as a co-stimulant. Cells were incubated for 15 h at 37 °C in a humidified atmosphere containing 5 % CO_2_. After 1 h, 10 µg/ml brefeldin A was added to the culture to stop Golgi transport. After incubation, cells were harvested and NKT cells were assayed for cytokine production by staining with anti-IFNγ-PE/Cy7, anti-TNFα-FITC, anti-IL-4-PE and anti-IL-17A-BV421 antibodies. NKT cells were stained with anti-CD3-PerCP and anti-TCR Vα24-Jα18-APC. All antibodies were obtained from BioLegend. Flow cytometry was performed on a FACS LSRII instrument (BD Biosciences).

Supernatants of an intracellular cytokine assay from a representative donor were tested for 12 analytes (IL-2, IL-4, IL-5, IL-6, IL-9, IL-10, IL-17A, IL-17F, IL-21, IL-22, IFNγ and TNFα) using the multi-LEGENDplex™ analyte flow assay kit (BioLegend). Briefly, antibodies specific for the 12 analytes were conjugated to 12 different fluorescence-encoded beads. The beads were mixed with serum samples (diluted twofold), incubated with shaking for 2 h at room temperature, washed, and incubated for 1 h with a cocktail of 12 different biotinylated detection antibodies. Finally, streptavidin-PE was added, the samples were incubated for 30 min, and the beads were washed and analyzed.

### Generation of APCs from CD14^+^ monocytes

To generate APCs, PBMCs were labeled with BD IMag™ Anti-Human CD14 Magnetic Particles (BD Biosciences). Next, 1 × 10^6^ CD14^+^ monocytes were plated in 6-well plates in 5 ml of RPMI (10 % FCS, 1 % l-Glutamine, 1 % Pen/Strep) with 500 U/ml recombinant human IL-4 (Sigma) and 500 U/ml human GM-CSF (MACS Miltenyi Biotec). On days 3 and 6, half of the media was replaced with fresh media containing IL-4 and GM-CSF. Immature APCs were harvested on day 7 or 8 and used in in vitro NKT cell stimulation assays. APC purity was determined by flow cytometry after staining with anti-CD11c-BV421, anti-HLA-DR-PE and anti-CD14-AF700 antibodies. All antibodies were obtained from BioLegend. The negative PBMC fraction lacking CD14^+^ monocytes was frozen in liquid nitrogen prior to use.

### NKT/PBMC cell activation without or with the addition of separately generated autologous APCs

For the NKT/PBMC cell assay without APCs, total 5 × 10^6^ PBMCs/well were stimulated with 10 µg/ml αGalCer or 20 µg/ml *Eh*LPPG in 100 µl of RPMI 1640 (10 % FCS, 1 % l-Glutamine, 1 % Pen/Strep) and incubated for 48 h at 37 °C under 5 % CO_2_. For the NKT/PBMC cell assay using the resultant APCs, 1 × 10^5^ APCs/well were stimulated with 10 µg/ml αGalCer or 20 µg/ml *Eh*LPPG in 50 µl of RPMI 1640 (10 % FCS, 1 % l-Glutamine, 1 % Pen/Strep) and incubated for 3–4 h at 37 °C under 5 % CO_2_. Next, 5 × 10^6^ thawed PBMCs from the negative (CD14^+^ monocyte-depleted) PBMC fraction described above was added in 50 µl of RPMI 1640 to the stimulated APCs, and the mixed samples were incubated for 48 h at 37 °C under 5 % CO_2_. Both assays were run in duplicate. After 48 h, supernatants were collected and assayed for IFNγ using ELISA MAX™ Standard SET Human IFNγ (BioLegend).

### Enrichment of human NKT cells

Enrichment of NKT cells was performed as described previously by Watarai et al. [26]. In brief, 2 × 10^6^ PBMCs were plated in 24-well plates in 1 ml of RPMI (10 % FCS, 1 % l-Glutamine, 1 % Pen/Strep) and specifically stimulated with either 100 ng/ml αGalCer or 400 ng/ml *Eh*LPPG to expand NKT cells at 37 °C, 5 % CO_2_. To encourage proliferation and maturation of T cells, 20 U/ml recombinant human IL-2 (rh-IL-2) (Cell Sciences) was added to all wells. rh-IL-2 alone was used as a negative control. Every 3–4 days, half of the media was replaced with fresh media containing 20 U/ml rh-IL-2. On day 9, 5 × 10^4^ APCs stimulated for 24 h with recombinant human IL-4, GM-CSF and αGalCer or *Eh*LPPG, respectively, were added to the expanding cells. Success of expansion was determined on day 0, 8 and 16 via flow cytometry to detect total NKT cells and NKT cell subpopulations.

### Statistical analysis

NKT cell frequencies and cytokine production were compared between women and men by Mann–Whitney *U* test. Comparisons of the cytokine production in stimulant-responding men and women (responders) and of NKT cell subsets following expansion were performed by unpaired Student’s *t* test. Differences were considered to be significant if *p* values were as follows: *p < 0.05; **p < 0.005; ***p < 0.0005.

## Results

### Women tend to have higher total and DN NKT cell frequencies than men

**Fig. 1:**
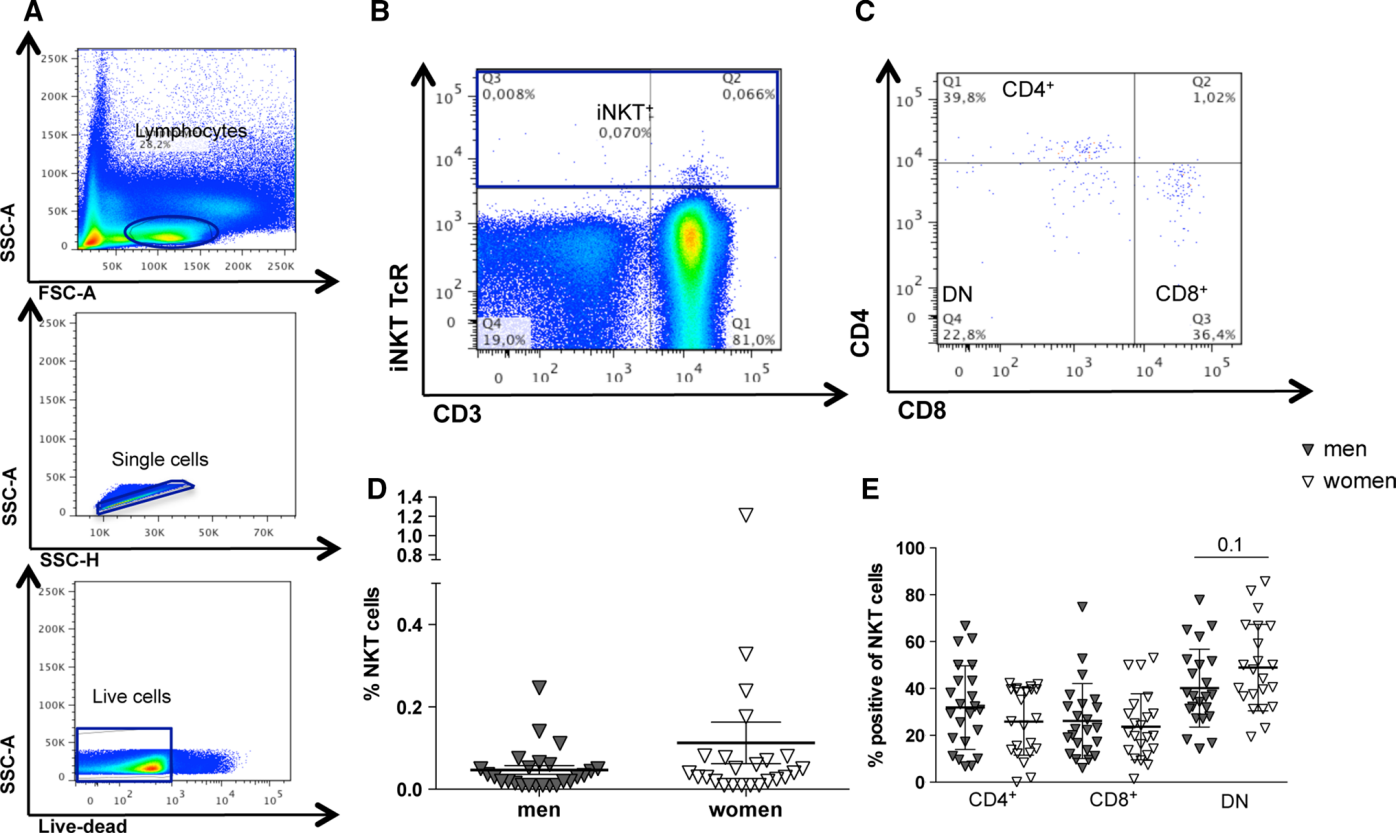
Frequencies of human peripheral blood NKT cells and NKT cell subpopulations in men and women. **a** PBMCs from healthy blood donors were analyzed by flow cytometry. Lymphocytes were gated in SSC and FSC, and single live cells were selected. **b** The frequency of NKT cells was determined by expression of CD3 and NKT TCR (Vα24-Jα18 TCR). **c** The frequencies of the NKT cell subpopulations were determined by expression of CD4 and CD8. **d** Sex-specific frequencies of NKT cells and **e** NKT cell subpopulations (means ± SEM; men, *n* = 23; women, *n* = 22; statistics: Mann–Whitney *U* test)

We used flow cytometry to analyze the frequencies of peripheral NKT cells and NKT cell subpopulation in 23 healthy male and 22 healthy female blood donors (Fig. 1a–e). The gating strategy is shown in Fig. 1a–c. After gating on lymphocytes, single cells were gated in SSC-A/SSC-H and only live cells were analyzed. NKT cells positive for NKT TCR (Vα24-Jα18) and CD3 (Fig. 1b) were further divided into their subpopulations (CD4^+^, CD8^+^ or DN) (Fig. 1c). Frequencies of peripheral NKT cells varied between 0.01 and 1.18 %. The frequency of NKT cells was higher in women (0.11 % ± SD) than in men (0.05 % ± SD), but this difference was not statistically significant (*p* = 0.3) (Fig. 1d). Analysis of CD4^+^, CD8^+^ and DN NKT subpopulations did not significantly differ between the sexes (Fig. 1e). The frequencies of CD4^+^ NKT cells was slightly higher in men (31.4 %) than in women (25.8 %), whereas the frequency of CD8^+^ NKT cells did not differ between the sexes (men: 26.1 %; women: 23.7 %). DN NKT cells were more abundant in women (48.9 %) than in men (40.2 %) (*p* = 0.1).

### Comparison of intracellular cytokine production by total NKT cells from men and women

**Fig. 2:**
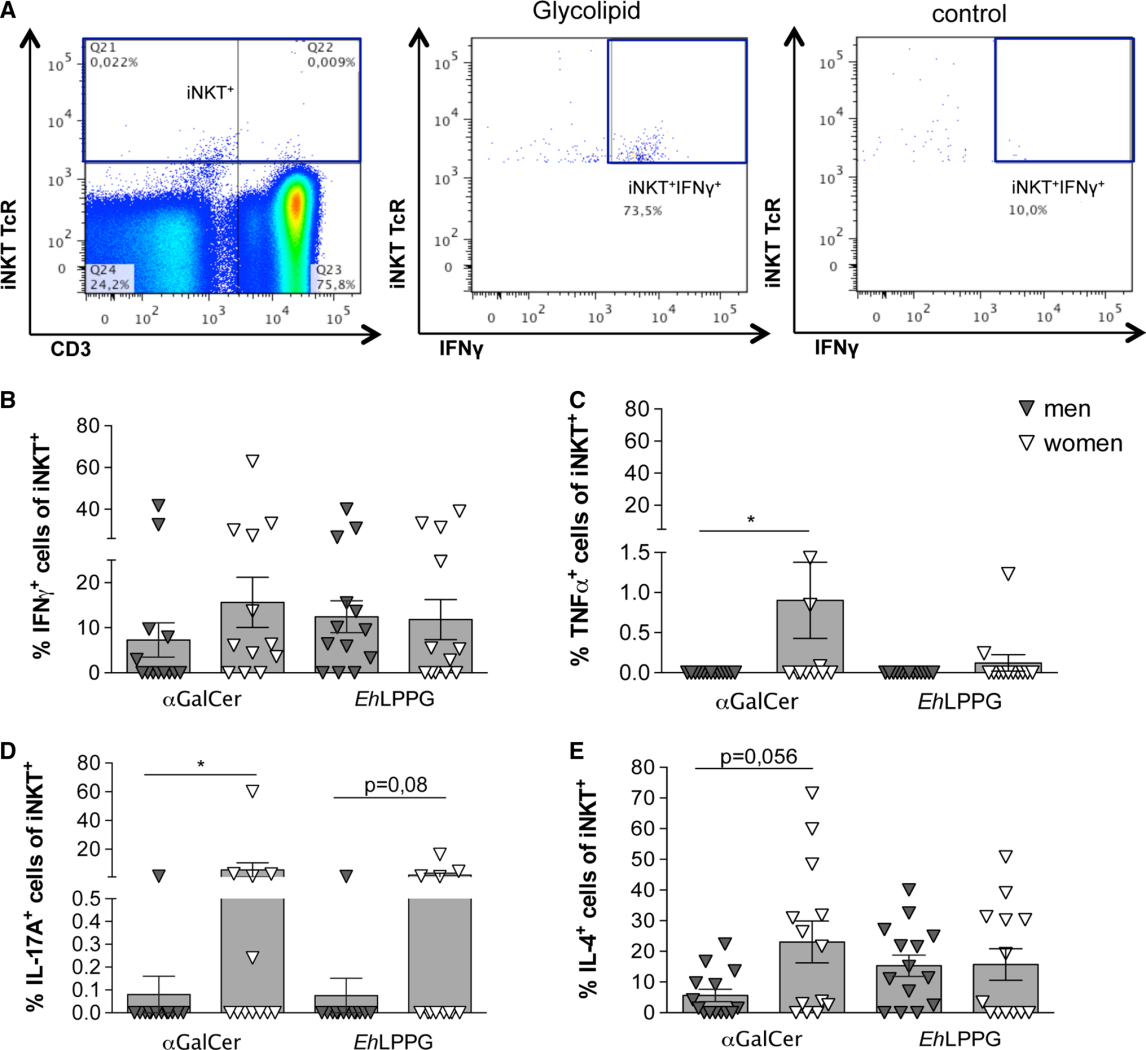
Comparison of intracellular cytokine production of peripheral NKT cells from men and women following stimulation with αGalCer and *Eh*LPPG. **a** Representative gating strategy for the analysis of intracellular cytokine production in peripheral blood NKT cell from men (*n* = 13) and women (*n* = 12). PBMCs were stimulated with αGalCer (1 µg/ml) or *Eh*LPPG (10 µg/ml) for 15 h. Percentage of NKT cells positive for intracellular **b** IFNγ, **c** TNFα, **d** IL-17A and **e** IL-4 production are shown (*bars* represent Means ± SEM; statistics: Mann–Whitney *U* test; *p < 0.05)

Following stimulation with αGalCer and *Eh*LPPG, we analyzed intracellular IFNγ, TNF, IL-17A and IL-4 production in 25 healthy blood donors (13 men and 12 women) by flow cytometry (Fig. 2a–e). The gating strategy is shown in Fig. 2a. Stimulation of NKT cells with the strong NKT cell ligand αGalCer resulted in a higher percentage of IFNγ^+^NKT cells in women (15.6 %) than in men (7.3 %) (Fig. 2b). Stimulation with *Eh*LPPG resulted in no sex-specific differences in the percentages of IFNγ^+^NKT cells (men: 12.4 %; women: 11.8 %) The proportion of TNFα^+^NKT cells was significantly higher in women (0.9 %) than in men (0.0 %) following αGalCer stimulation (*p* < 0.02), whereas *Eh*LPPG induced very few TNFα^+^NKT cells in either sex (Fig. 2c). Furthermore, αGalCer and *Eh*LPPG both induced higher percentages of IL-17A^+^NKT cells in women (αGalCer: 5.6 %; *Eh*LPPG: 1.9 %) than in men (αGalCer: 0.08 %; *Eh*LPPG: 0.07 %) (Fig. 2d); the effect of αGalCer was statistically significant (*p* < 0.03). NKT cells from women also exhibited a higher percentage of IL-4^+^ NKT cells after αGalCer stimulation compared to men (*p* < 0.056) while stimulation with *Eh*LPPG revealed no difference in the percentage of IL-4^+^ NKT cells between the sexes (women: 15.7 %; men: 15.3 %). Thus, αGalCer stimulation induced a higher percentage of cytokine-producing cells than *Eh*LPPG and significant sex-specific differences in production of TNF, IL-17A and IL-4.

### CD4^+^ NKT and DN NKT cells are the major cytokine-producing NKT subpopulations in both sexes

**Fig. 3:**
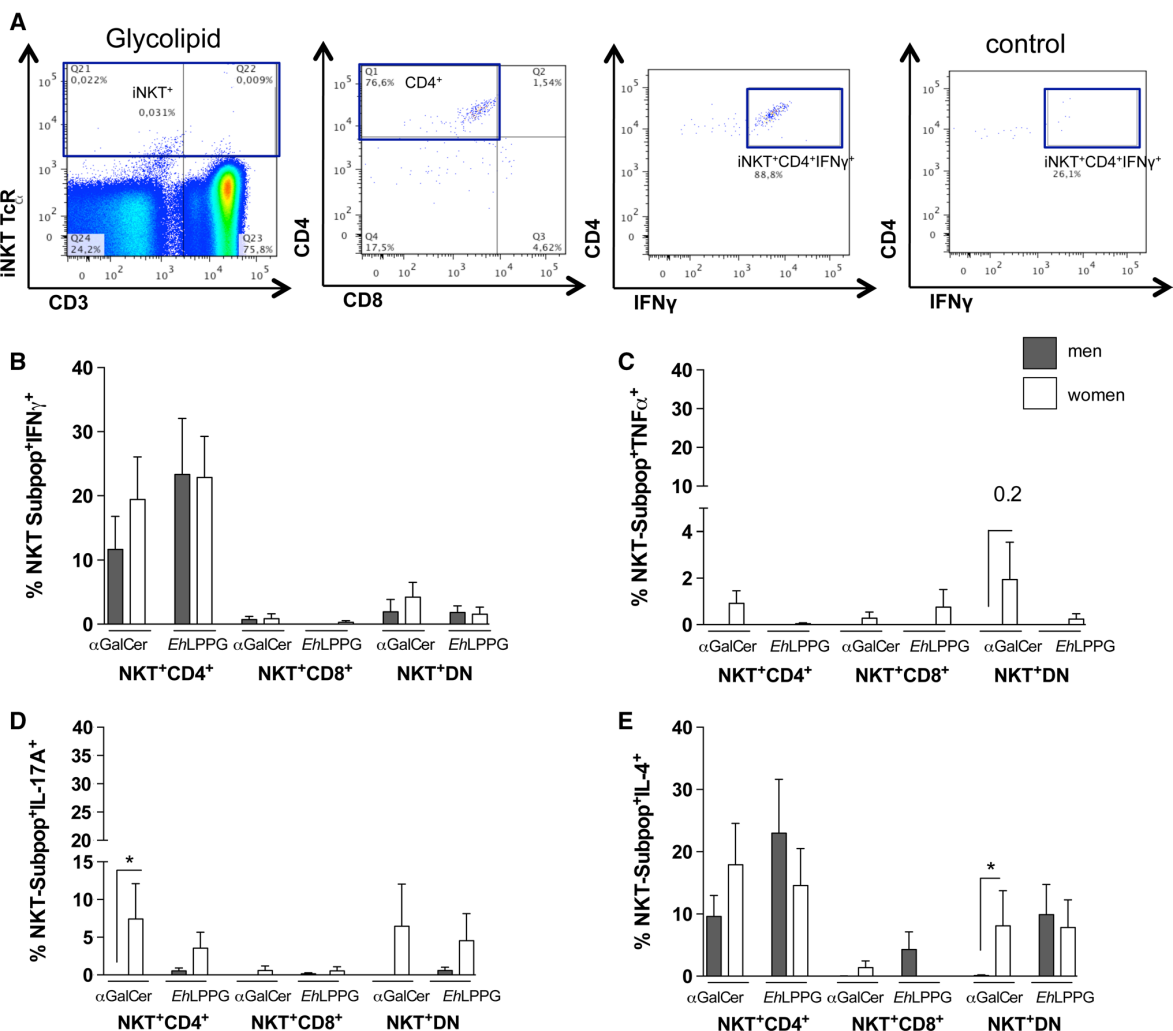
Intracellular cytokine production in peripheral NKT cell subpopulations from men and women following stimulation with αGalCer and *Eh*LPPG. **a** Representative gating strategy for the analysis of intracellular cytokine production in peripheral blood NKT cell subpopulations from men (*n* = 13) and women (*n* = 12). PBMCs were stimulated with αGalCer (1 µg/ml) or *Eh*LPPG (10 µg/ml) for 15 h. Percentage of NKT cell subpopulations positive for **b** IFNγ, **c** TNFα, **d** IL-17A and **e** IL-4 NKT are shown (*bars* represent Means ± SEM; statistics: Mann–Whitney *U* test; *p < 0.05)

Next, we sought to determine which NKT cell subpopulations are responsible for the observed cytokine production in men and women (Fig. 3a–e). Figure 3a shows the gating strategy used to determine NKT cell subpopulations. We analyzed cytokine production by CD4^+^, CD8^+^, and DN NKT cell subpopulations after stimulation with αGalCer or *Eh*LPPG. Following stimulation with either ligand, CD4^+^ NKT cells were the primary producers for IFNγ production with no significant difference between men and women although the percentage was higher in women after αGalCer stimulation (19.4 %; men: 11.6 %) (Fig. 3b). CD8^+^ and DN NKT cells contributed only weakly to IFNγ production. TNF^+^ NKT cells were very scarce and were detectable only following αGalCer stimulation in DN NKT cells from women (Fig. 3c).

IL-17A production was observed primarily following αGalCer stimulation of CD4^+^ and DN NKT cells, and little IL-17A was produced following stimulation with *Eh*LPPG of DN NKT cells. The proportion of CD4^+^ NKT cells was significantly higher in women than in men (*p* = 0.03; Fig. 3d).

CD4^+^ NKT cells were the main cell producers of IL-4 following αGalCer and *Eh*LPPG stimulation, and these cells were more abundant in women following αGalCer stimulation (women: 17.9 %; men: 9.5 %; Fig. 3e). *Eh*LPPG induced a higher percentage of CD4^+^IL-4^+^ NKT cells in men (men: 22.9 %; women: 14.5 %). Women had a significantly higher percentage of DN IL-4^+^ NKT cells (8.1 %; *p = 0.03) than men, whereas *Eh*LPPG stimulation induced no sex-specific difference in the percentage of DN IL-4^+^ NKT cells.

In summary, these data revealed significant sex-specific differences in IL-17A and IL-4 production in CD4^+^ and DN NKT cells, respectively. The CD4^+^ and DN NKT subpopulations were the main cytokine producers in women and men.

### Sex-specific IFNγ production by bystander cells following αGalCer stimulation and addition of autologous APCs

**Fig. 4:**
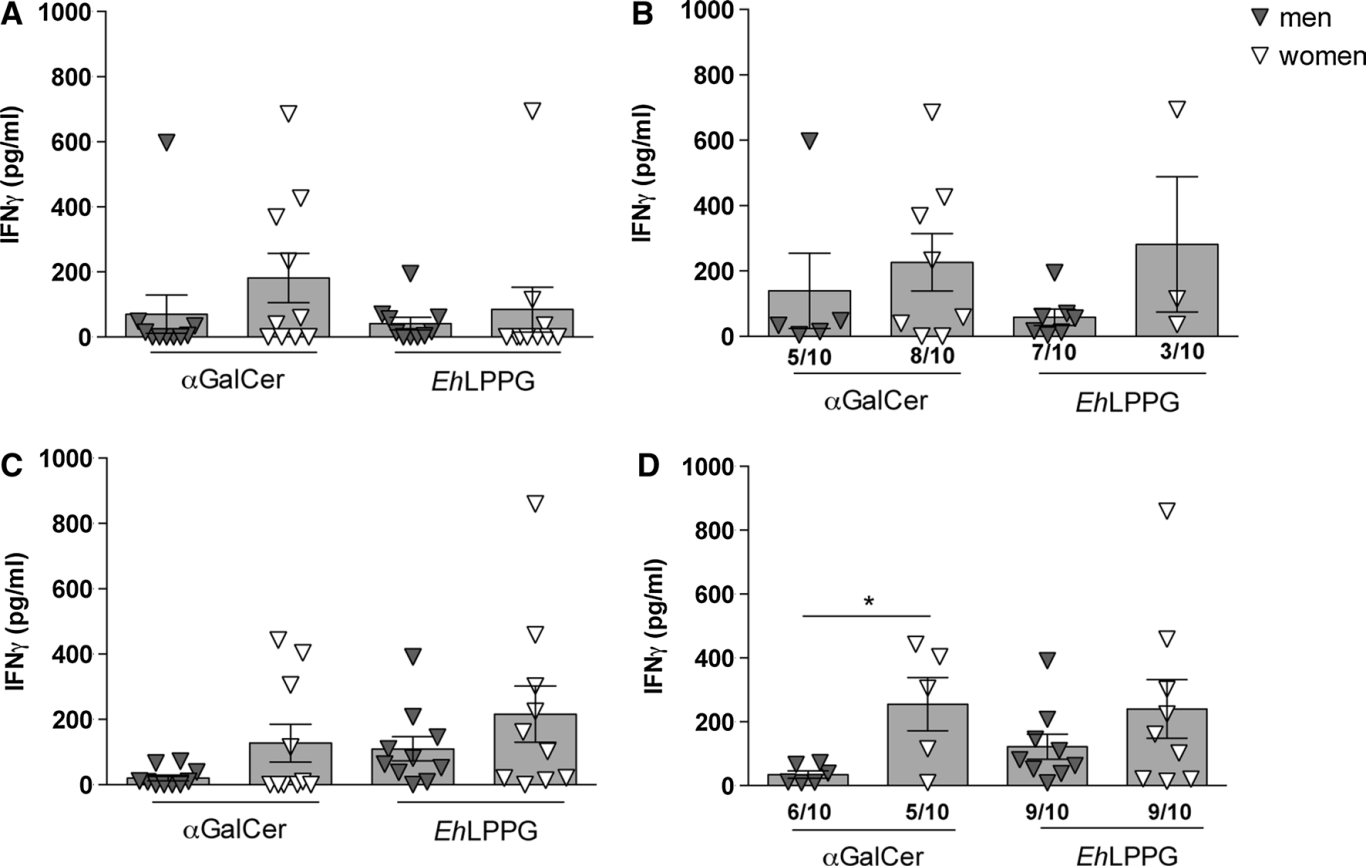
Influence of autologous APCs on IFNγ production by human PBMCs. IFNγ production by **a** PBMCs from male (*n* = 10) and female (*n* = 10) blood donors stimulated with αGalCer (1 µg/ml) or *Eh*LPPG (20 µg/ml) for 48 h. **b** IFNγ production in the same donors excluding samples that did not respond to the indicated stimuli (male, *n* = 5–7; female, *n* = 3–8). IFNγ production by **c** PBMCs from male (*n* = 10) and female (*n* = 10) blood donors co-cultured 48 h with autologous, in vitro generated APCs pulsed with αGalCer (1 µg/ml) or *Eh*LPPG (20 µg/ml). **d** IFNγ production of the same blood donors excluding samples that did not respond to the stimulation by pre-pulsed APCs (male, *n* = 6–9; female, *n* = 5–9). Cytokine production was measured by ELISA (means ± SEM; men, *n* = 10; women, *n* = 10; statistics: unpaired Student’s *t* test; *p < 0.05)

To investigate sex-specific differences in IFNγ production by PBMCs following NKT cell activation, we stimulated PBMCs (Fig. 4a, b) with αGalCer and *Eh*LPPG either alone or in the presence of autologous APCs (Fig. 4c, d). Several donors did not respond to stimulation with αGalCer and *Eh*LPPG by producing IFNγ above the level of the corresponding medium control. Nonetheless, even including the non-responding donors, we observed slightly higher IFNγ production by PBMCs in women, irrespective of the presence or absence of APCs (Fig. 4a, c). When the non-responders were excluded, this difference became more pronounced (Fig. 4b) and the sex-specific difference became statistically significant following stimulation with αGalCer in the presence of APCs (*p* < 0.05, Fig. 4d). As described previously [1], *Eh*LPPG must be processed by APCs to exert optimal NKT cell-specific activity. This is reflected in the observation that more subjects responded following the addition of independently generated homologous APCs (Fig. 4b: men: 7/10 and women: 3/10; Fig. 4d: men: 9/10 and women: 9/10; men PBMC vs. men PBMC with APCs; women PBMC vs. women PBMC with APC). In contrast, we observed no difference between responding and non-responding individuals when αGalCer was used as the stimulus. Thus, PBMCs contribute to sex-specific IFNγ production, and APCs increase the stimulatory activity of *Eh*LPPG.

### Expression of multiple cytokines is induced in PBMCs following αGalCer and EhLPPG stimulation

**Fig. 5:**
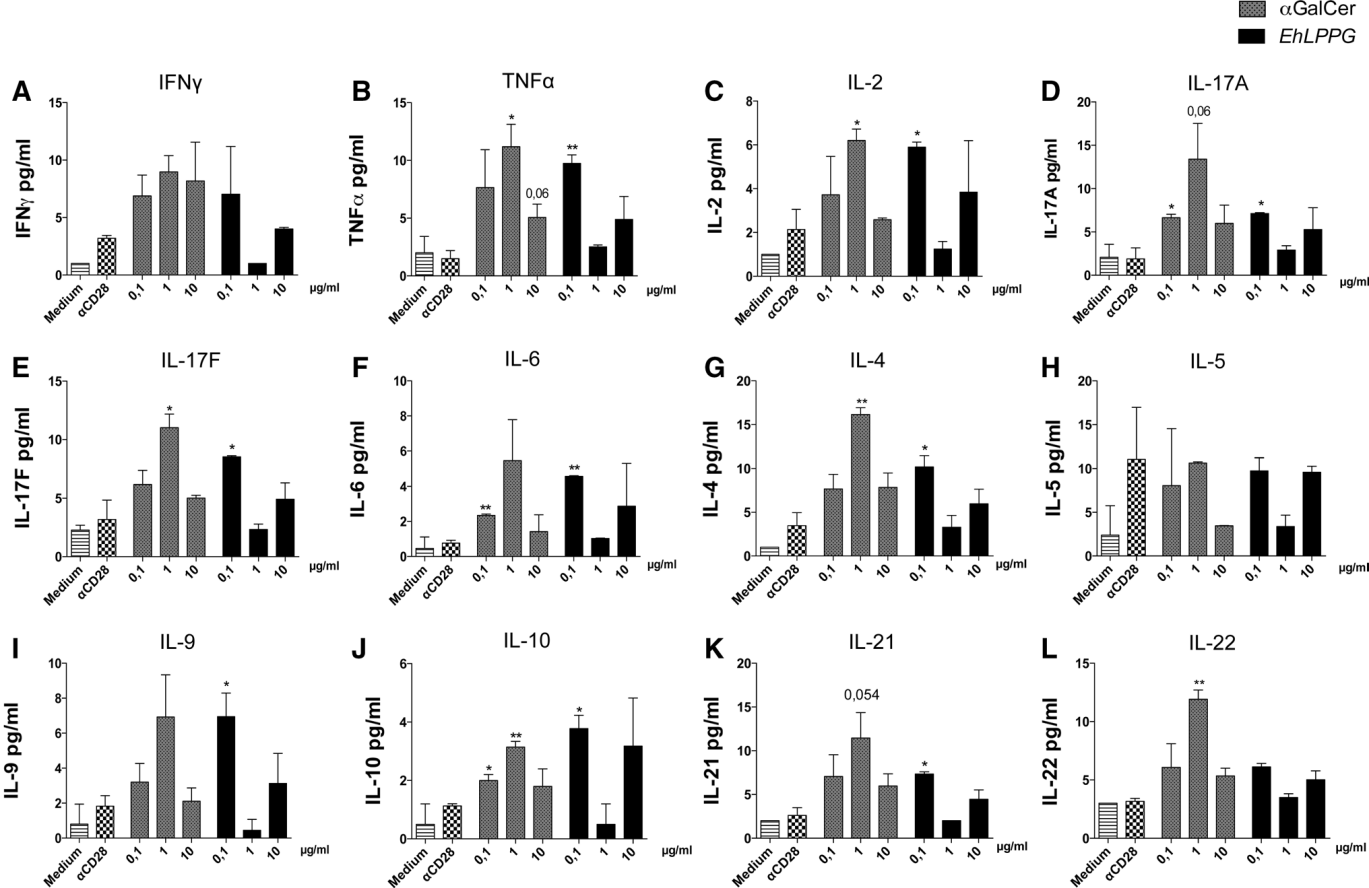
Spectrum of cytokine production by PBMCs stimulated with αGalCer and *Eh*LPPG. Cytokine profile in the supernatant of PBMCs from a female blood donor stimulated for 15 h with concentrations of 0.1, 1.0 and 10 µg/ml αGalCer or *Eh*LPPG. LEGENDplex™ Cytometric Bead Assay (BioLegend) was used to determine the concentrations of **a** IFNγ, **b** TNFα, **c** IL-2, **d** IL-17A, **e** IL-17F, **f** IL-6, **g** IL-4, **h** IL-5, **i** IL-9, **j** IL-10, **k** IL-21 and **l** IL-22 (means ± SEM; *n* = 1; statistics: unpaired Student’s *t* test; *p < 0.05; **p < 0.01)

To extend the analysis of the cytokine repertoire induced by αGalCer and *Eh*LPPG, we performed a Cytometric Bead Assay (LEGENDplex™) using the supernatants of PBMCs stimulated with different amounts of αGalCer and *Eh*LPPG from one female donor. Supernatant from unstimulated PBMCs and PBMCs stimulated with anti-CD28 were used as controls (Fig. 5a–l). Depending on the concentration of the stimulus agent, most of the 12 cytokines were detectable at significant levels following stimulation with either αGalCer or *Eh*LPPG. Following stimulation with 0.1 µg–1.0 µg/ml αGalCer, levels of TNF, IL-2, IL-17A, IL-17F, IL-6, IL-4, IL-10 and IL-22 were significantly elevated (*p* < 0.05–0.01); IFNγ, IL-9 and IL-21 levels were elevated, but not significantly; and IL-5 was not detectable at all. Higher concentrations of αGalCer decreased cytokine production. Following stimulation with 0.1 µg/ml *Eh*LPPG, levels of TNF, IL-2, IL-17A, IL-17F, IL-6, IL-4, IL-9 and IL-21 were significantly elevated (*p* < 0.05–0.01). IFNγ and IL-21 levels were elevated, and IL-5 was not detectable. Increased concentrations of *Eh*LPPG did not lead to an increase in cytokine levels.

In summary, stimulation with αGalCer and *Eh*LPPG resulted in the expression of a variety of Th1- and Th2 cytokines in a reverse dose-dependent manner.

### The ratio of NKT cell subpopulations in enriched NKT cells is sex specific and stimulus dependent

**Fig. 6:**
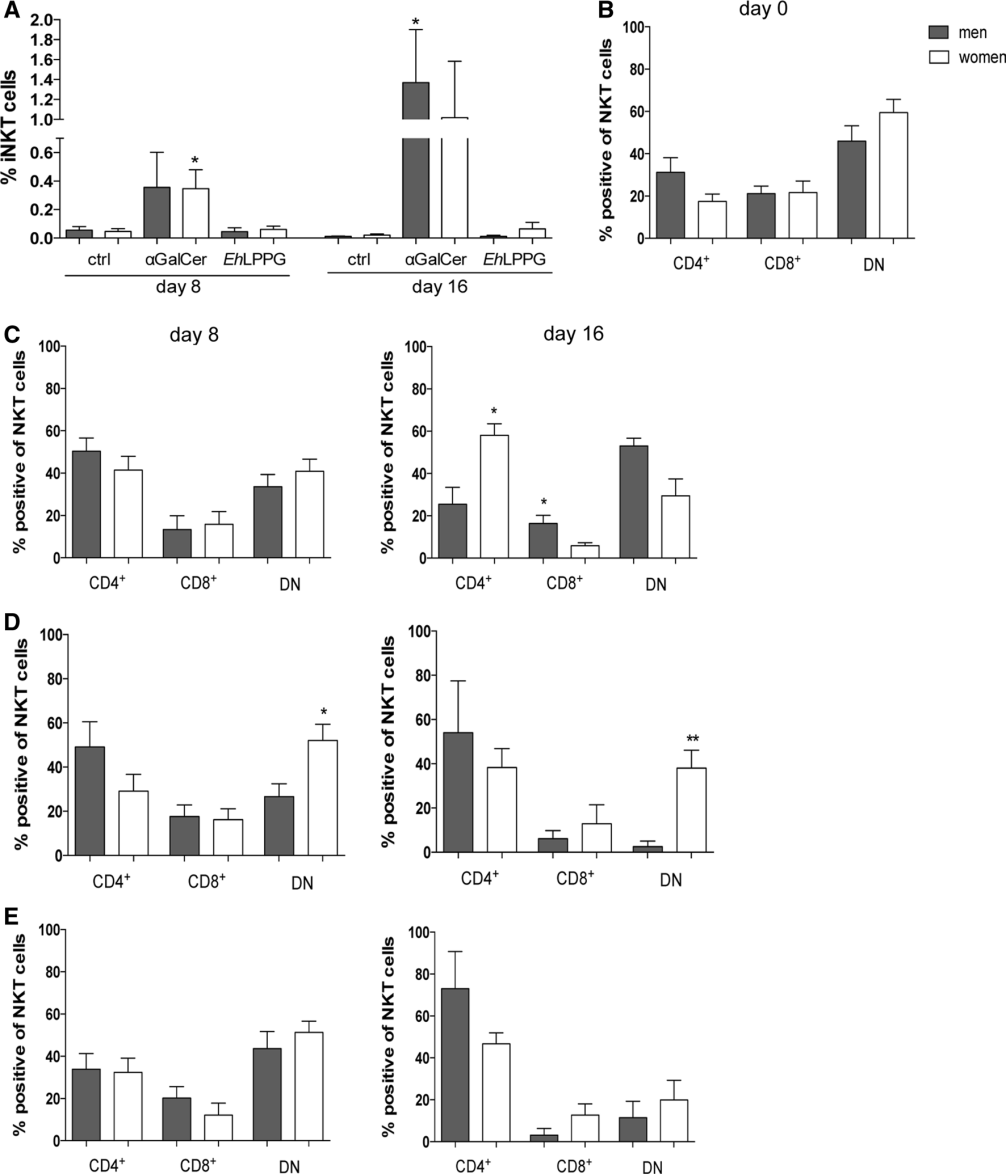
The ratio of NKT cell subpopulations in enriched NKT cells is sex specific and dependent on stimulant. a Frequency of NKT cells on day 8 and day 16 after enrichment with 20U/ml rh-IL-2, 100 ng/ml αGalCer and 400 ng/ml *Eh*LPPG, respectively. **b** NKT cell subpopulations in men and women on days 0, 8 and 16 in the presence of **c** αGalCer, **d** *Eh*LPPG or **e** rh-IL-2 alone (control) (means ± SEM; men, *n* = 10; women, *n* = 10; statistics: unpaired Student’s *t* test; *p < 0.05)

Continuous stimulation of the invariant TCR with αGalCer can lead to an enrichment of NKT cells [26]. Therefore, we investigated whether continuous stimulation with αGalCer and *Eh*LPPG over 16 days would increase the total NKT cell number and influence the ratio of NKT cell subpopulations in a sex-specific manner (Fig. 6). We stimulated PBMCs from 10 men and 10 women for 16 days with 100 ng/ml αGalCer and 400 ng/ml *Eh*LPPG. Autologous APCs were added on day 9. Interestingly, *Eh*LPPG stimulation did not result in an increase of the total NKT cell number, whereas αGalCer treatment led to increases on day 8 (0.36 % in men and 0.35 % in women; *p* < 0.05) and day 16 (1.4 % in men (*p* < 0.015) and 1.0 % in women) (Fig. 6a). Analysis of the NKT cell subpopulations that were enriched over time in the presence of αGalCer revealed similar proportions of CD4^+^, CD8^+^ and DN NKT cells in men and women from day 0 up to day 8 (Fig. 6b, c). On day 16, more CD4^+^ NKT cells were present in women (*p* < 0.015) than in men, whereas CD8^+^ NKT cells (*p* < 0.043) and DN NKT cells were more abundant in men (Fig. 6c). By contrast, over time *Eh*LPPG treatment caused a significant shift toward higher DN NKT cells in women compared to men (day 8, *p* < 0.015; day 16, *p* < 0.006), Fig. 6d). This sex-specific shift did not occur when PBMCs were cultured without an NKT cell-stimulating agent (Fig. 6e); under these conditions, we observed a tendency toward a sex-independent increase in the proportion of CD4^+^ NKT cells.

In summary, we found that αGalCer, but not *Eh*LPPG, increased NKT cell numbers during long-term culture of PBMCs and both stimuli induced a sex-specific shift in the relative proportions of the NKT cell subpopulations.

## Discussion

Immune responses differ between the sexes, and differences in the frequency and activation of immune cells could contribute to the observed sex-specific differences in infectious and autoimmune diseases. NKT cells are important immune modulators involved in early control of an infection [14, 27]. As shown recently, the cytokine profile of these cells is modulated by sex hormones; specifically, estrogens increase and testosterone decrease the secretion of the proinflammatory cytokine IFNγ [24, 25]. In this study, we characterized the sex-specific differences in NKT cell frequency and cytokine production in response to the strong NKT cell activator αGalCer and the moderate NKT cell activator *Eh*LPPG.

Several studies report that the frequency of NKT cells is higher in women than in men [11–13]. We also observed higher NKT cell numbers in women, although the differences were not statistically significant. By contrast, other studies observed no correlation between sex and NKT cell frequency [19, 28]. Various studies report different ratios of NKT cell subpopulations in each sex. We observed slightly higher percentages of CD4^+^ and CD8^+^ NKT cells in men and higher percentages of DN NKT cells in women. Two other studies also observed higher CD4^+^ NKT cell numbers in men [11, 13], whereas another study observed the opposite distribution in this subpopulation and more CD8^+^ and DN NKT cells in men [19]. Consistent with our results, Kee et al. [13] also observed a higher frequency of DN NKT cells in the peripheral blood of women. These differences in the observed frequencies of NKT cells and their subpopulations may be due to the quantity or age of the subjects in each study. Consistent with this idea, several studies showed that NKT cell frequency significantly decreases with age [28–31]. Moreover, the sex-specific difference in the abundance of NKT cells may have an impact on diseases with sexual dimorphism. Human DN NKT cells mainly produce Th1 cytokines [32]. Therefore, the elevated percentage of DN NKT cells in women suggests that production of protective IFNγ during infection will be higher in women than in men. Parasite-induced amebic liver abscess (ALA) has a strong sex bias toward men, and a mouse model of the disease exhibits a similar preference toward males; however, the resistance of female mice is abolished in mice lacking NKT cells and IFNγ [1].

Sex-specific differences in cytokine secretion were documented for several immune cell populations. Phytohemagglutinin-stimulated lymphocytes from male blood donors produced higher levels of IFNγ and IL-2 and lower levels of IL-4 and IL-10 than those from females [33]. Additionally, following LPS stimulation monocytes from men produce more TNFα, IL-1β and IL-12 than those from women [2]. In the mouse model of ALA, NKT cells from female mice produce more IFNγ than NKT cells from male mice [25] and female mice have higher serum IFNγ levels following αGalCer treatment [24]. In part due to the extremely low number of circulating NKT cells in men (0.001–0.1 %), little is known about sex-specific differences in the cytokine production by human NKT cells. In this study, however, we were able to analyze intracellular cytokine production by peripheral NKT cells by flow cytometry. We found that stimulation with the strong NKT cell activator αGalCer resulted in significantly more TNFα and IL-17A-positive NKT cells and a trend toward more IFNγ and IL-4-positive NKT cells in female blood donors. By contrast, *Eh*LPPG stimulation induced no sex difference in the production of IFNγ, TNFα or IL-4 by NKT cells and a non-statistically significant increase in the percentage of IL-17A-positive NKT cells in women. Among the three NKT cell subpopulations, CD4^+^ and DN NKT cells were the major cytokine producers. In particular, CD4^+^ and DN NKT cells from female blood donors produced significantly more IL-17A after αGalCer stimulation. In addition, more IL-4^+^DN NKT cells were present in samples from female blood donors. *Eh*LPPG induced no significant sex difference in the cytokine production by NKT cells, although a tendency toward sex-specific differences could be observed for IL-17A^+^CD4^+^, IL-17A^+^DN and IL-4^+^CD4^+^ NKT cells. By contrast, mitogen stimulation of sorted human NKTs resulted in higher proportions of IFNγ-, TNFα- and MIP-1-α-producing NKT cells in men than in women, but no difference in IL-4 production by NKT cells [19].

In another study using in vitro expanded human NKT cells, αGalCer stimulation induced Th1 and Th2 cytokines in all three NKT subpopulations, but cytokine production was higher in CD8^+^ NKT cells than in CD4^+^ and DN NKT cells [20]. Analysis of cytokine production by PBMCs following specific NKT cell activation by αGalCer or *Eh*LPPG revealed that non-NKT cells also contribute to cytokine production, putatively via indirect activation, which might reflect the immunoregulatory properties of NKT cells. In this study, we observed induction of a variety of cytokines, including the Th1 cytokines IFNγ, TNF, IL-17 A/F and IL-2 and the Th2 cytokines IL-4 and IL-10, as well as a strong induction of the pluripotent cytokine IL-6.

This observation might indicate that NKT cells express a sex-specific cytokine repertoire that differs depending on the used stimulant. A specific ligand like αGalCer may induce a gene expression pattern that involves sex hormone receptors, leading to a sex-dependent cytokine production. By contrast, a less specific mitogenic ligand like phytohaemagglutinin may induce more general regulation of immunity-related gene expression and might reflect the chromosomal repertoire of possible cytokine production.

The nature of the stimulus may also be important for the sex specificity of APCs such as plasmacytoid DCs (pDCs), which influence the subsequent cytokine production in responding immune cells. For example, pDCs from women produce higher levels of IFNα following ligation of the TLR7-agonist imiquimod, a heterocyclic amine, than pDCs from men [34]. Moreover, immune cells from female mice and rats express higher levels of pathogen recognition receptors, such as TLR2, 3 and 4, and mount a more efficient immune response against specific pathogens than those from male rats [35]. In our assays, we also observed a sex-specific influence of APCs on immune activation. Although sex-specific differences in cytokine production were also observed in the absence of separately generated autologous APCs, addition of these cells revealed more pronounced differences between the sexes.

The stimulus could also modulate the ratio of NKT cell subpopulations in a sex-dependent manner. Along these lines, we found that αGalCer induced strong NKT cell enrichment and favored the development of higher proportions of CD4^+^ NKT cells in PBMC cultures from female blood donors and higher proportions of CD8^+^ and DN NKT cells in the cultures from male blood donors. *Eh*LPPG induced no enrichment of NKT cells, but still influenced the ratio of the NKT cell subpopulation over the course of long-term culture. Under these conditions, we observed a shift toward the CD4^+^ NKT cell subpopulation in men and CD4^+^ and DN NKT cell subpopulation in women.

Therefore, because *Eh*LPPG facilitates NKT cell activation through engagement of TLR2, TLR4 and TLR6 [1], we hypothesize that *Eh*LPPG provides a survival signal that is sufficient to alter the ratio of the NKT cell subpopulations.

In conclusion, we showed that women tend to have higher NKT cell frequencies than men and that their NKT cells produce significantly more TNFα and IL-17A following αGalCer stimulation. Moreover, a strong stimulus like αGalCer is more likely to induce distinct and significant sex-specific differences in the cytokine profile of human NKT cells than a more moderate or weaker stimulus such *Eh*LPPG. Some stimuli may induce sex differences and a sex-specific outcome of a disease, whereas others may induce similar immune responses in both men and women. Sex hormones and X-chromosome-linked immune regulatory genes are thought to be major players in sex-specific immune responses, but the precise details of their regulatory biology and interactions remain unclear. Although some immune cell subsets exhibit sex-specific differences in frequency, the quality of the mounted immune response is likely to be far more important for sexual dimorphism in disease than the quantities of immune cells involved.

## Abbreviations

αGalCer: αGalactosylceramide
*Eh*LPPG: *Entamoeba histolytica* lipopeptidephosphoglycan
DN: Double negative
